# Interfacial Penetration Drives Anomalous Domain Spacing in Strongly Segregated Linear-Bottlebrush Copolymers

**DOI:** 10.1021/acsmacrolett.6c00296

**Published:** 2026-08-20

**Authors:** Baiqiang Huang, Haobo Wang, Patryk Wąsik, Eliot Gann, Li-Heng Cai

**Affiliations:** † Department of Materials Science and Engineering, 2358 University of Virginia, Charlottesville, Virginia 22904, United States; ‡ National Synchrotron Light Source II, 8099 Brookhaven National Laboratory, Upton, New York 11973, United States; § Department of Chemical Engineering, University of Virginia, Charlottesville, Virginia 22904, United States; ∥ Department of Biomedical Engineering, University of Virginia, Charlottesville, Virginia 22904, United States; ⊥ Department of Chemistry, University of Virginia, Charlottesville, Virginia 22904, United States

## Abstract

Domain spacing in block copolymers is typically limited by polymer contour length. Here, we report anomalously large domain spacing in strongly segregated linear–bottlebrush diblock copolymers. Using transmission electron microscopy and small-angle X-ray scattering, we show that the bridging distance between neighboring linear domains exceeds the bottlebrush backbone contour length. Notably, the excess length matches that previously observed in linear–bottlebrush–linear triblock copolymers, despite the inability of diblocks to form physical networks. This agreement rules out network formation as the origin of the anomaly. We propose that the penetration of linear segments into the bottlebrush domain relieves side chain packing frustration and increases the effective bottlebrush domain size. These findings reveal a previously unrecognized mechanism for generating domain spacing beyond contour-length limits and suggest that molecular architecture can be used to program large length scales in nanostructured soft materials.

## Graphical abstract

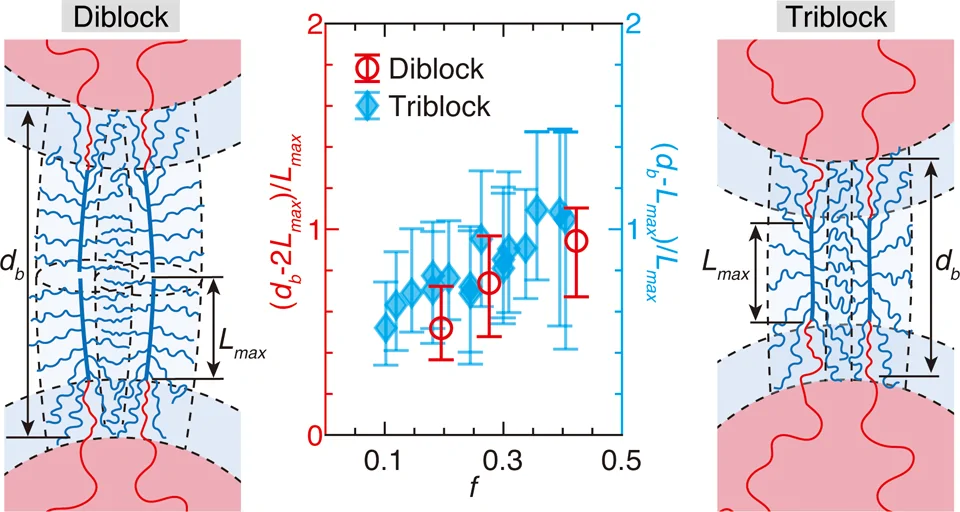

A block copolymer consists of chemically distinct polymer chains linked by covalent bonds. When the blocks are incompatible, they undergo microphase separation and self-assemble into periodic nanostructures such as lamellae, cylinders, and spheres.^1,2^ This behavior underpins diverse applications ranging from nanolithography,^3^ porous structures for filtration and separation,^4^ and drug delivery.^5^ For AB diblock or ABA triblock copolymers composed of flexible linear polymers, self-assembly is determined mainly by the composition *f* and the segregation strength χ*N*, where χ is the Flory–Huggins interaction parameter and *N* is the total number of Kuhn monomers per copolymer.^6^ In the strongly segregated limit (χ*N* ≫ 10), the interfacial thickness δ, over which the composition transitions from nearly pure A to nearly pure B domains, is independent of *N* and scales as δ ∼ *b*χ^–1/2^, where *b* is the statistical segment or Kuhn length.^7^ Because χ > 0.1, δ is on the order of Kuhn length *b* and therefore much smaller than the domain spacing, *d*_*b*_ ∼ *bN* ^2/3^.^8^

Replacing a linear block with a bottlebrush polymer introduces pronounced conformational asymmetry. A bottlebrush polymer consists of a linear backbone densely grafted with side chains.^9^ Because of strong steric repulsion among highly overlapped side chains, the bottlebrush is bulky and relatively stiff compared to linear polymers.^10,11^ As a result, the self-assembly of linear-bottlebrush block copolymers depends not only on *f* and χ*N*, but also on architectural parameters, including side chain length, grafting density, and backbone length.^12–18^

In our previous work, we studied the self-assembly of strongly segregated linear–bottlebrush–linear (LBL) triblock copolymers.^19–21^ We discovered that the bridging distance between neighboring linear domains exceeds twice the contour length of the bottlebrush backbone, which is the maximum span of an isolated bottlebrush. This observation challenges the classical understanding in which the domain spacing cannot be greater than the polymer contour length. We proposed that the interfacial tension drives penetration of an individual linear end into the bottlebrush domain, effectively increasing its size. Importantly, the penetration depth is approximately 10 nm, which is remarkably larger than the conventionally thought interfacial thickness (∼1 nm).^7^ In these systems, the linear blocks are poly­(benzyl methacrylate) (PBnMA) and the middle block is bottlebrush polydimethylsiloxane (PDMS), which can form strong physical networks that remain stable up to high temperature (200 °C).^21,22^ Such network formation may hinder equilibrium during self-assembly, even under thermal and solvent annealing, and thus influence the observed domain spacing. This raises a key question: Does the anomalous domain spacing persist in a system that cannot form physical networks?

Here, we address this question by systematically investigating linear–bottlebrush (LB) diblock copolymers. Unlike LBL triblock copolymers, diblock copolymers have only one linear block and cannot form physical networks. Using transmission electron microscopy (TEM) and small-angle X-ray scattering (SAXS), we characterize the resulting microstructures and quantify the characteristic length scales. We find that the bridging distance also exceeds the contour length of the bottlebrush backbone. Notably, the excess length is the same as that observed in triblocks, consistent with linear chain penetration from one end in diblocks versus both ends in triblocks. These results rule out network formation as the origin of the anomalously large domain spacing and strongly support interfacial chain penetration as the governing mechanism. This work establishes interfacial chain penetration as a route to achieve domain spacing beyond contour-length limits in architecturally complex polymers, offering a new strategy to program large length scales in nanostructured soft materials.

We design and synthesize LB diblock copolymers using our established approach (Figure 1a).^21,22^ The bottlebrush block consists of 40 methacrylate-terminated PDMS side chains with a molecular weight (MW) of 5 kg/mol and a grafting distance *l* = 0.254 nm (Figure 1b). Such architecture yields a bulky macromolecule with a cross-section *b*_*e*_ ≈ 13 nm, comparable to the backbone contour length $L_{\max}\approx 10\mathrm{nm}$ . To avoid architectural variations, we first synthesize a relatively large amount of bottlebrush with precisely 40 PDMS side chains and then use the same bottlebrush as a macroinitiator to grow PBnMA linear blocks of various molecular weights. Gel permeation chromatography (GPC) of the bottlebrush shows a relatively narrow, symmetric peak with a polydispersity index (PDI) of 1.20 (dashed line, Figure 1c). For all diblock copolymers, we use ^1^H NMR to determine average MW (see Supporting Information NMR spectra) and GPC to characterize the sample quality. As expected, the GPC peak retention time decreases as the MW of the end blocks increases (solid lines, Figure 1c). Controlled polymerization is further validated by the exponential relationship between molecular weight *M* and retention time *t* (Figure 1d). The molecular parameters of all polymers are summarized in Table 1.

**Figure 1:**
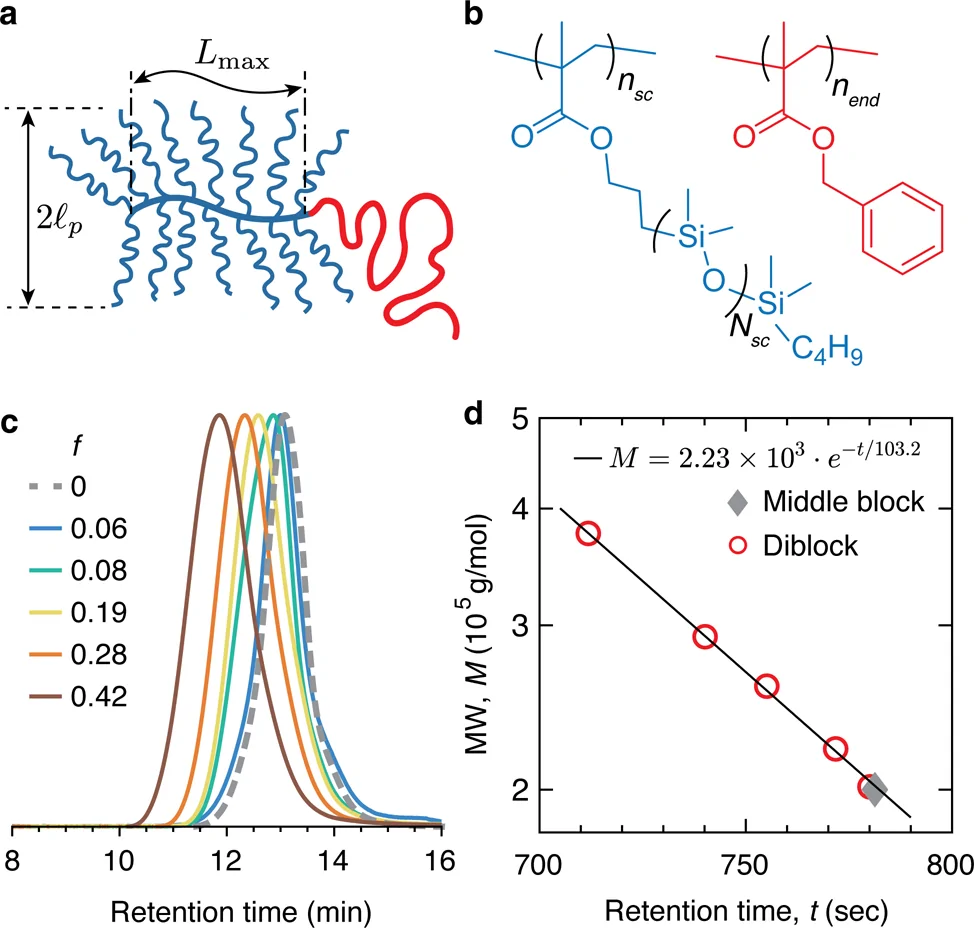
Molecular design and synthesis of linear–bottlebrush (LB) diblock copolymers. (a) Schematic of an LB diblock copolymer. The bottlebrush block has a contour length $L_{\max}$ and a persistence length $l_{p}\approx L_{\max}/2$ , corresponding to a flexibility $\kappa \equiv L_{\max}/\left(2l_{p}\right)\approx 1$ . The linear block (red) is covalently linked to one end of the bottlebrush. (b) Chemical structures of the LB diblock copolymer. The bottlebrush block consists of polydimethylsiloxane (PDMS) side chains (blue), where *N*_sc_ is the degree of polymerization (DP) per side chain, and *n*_sc_ is the number of side chains per bottlebrush. The linear end block is poly­(benzyl methacrylate) (PBnMA, red), where *n*_end_ is the DP of the end block. (c) GPC traces of the bottlebrush PDMS block (dashed line, *f* = 0) and all LB diblock copolymers (solid lines), where *f* is the volume fraction of the linear PBnMA block. (d) The molecular weight *M* of all diblock copolymers decreases exponentially with the retention time *t*. Gray diamonds represent the bottlebrush block; red circles represent the diblock copolymers.

**Table 1:** Molecular Parameters of LB Diblock Polymers^a^

							microstructure				
	middle block			diblock			q* (nm^–1^)			d* (nm)	*D* (nm)
sample	*M*_sc_ (kDa)	*n*_sc_	PDI	*n*_end_	*f*	PDI	peak	upper	lower	peak	TEM
S0	5	40	1.20	NA	NA	NA	0.964	0.858	1.070	6.5	NA
S1				8	0.01	1.21	0.964	0.858	1.070	6.5	NA
S2				120	0.08	1.28	0.204	0.162	0.246	30.8	NA
S3				330	0.19	1.30	0.152	0.131	0.173	41.3	22.6 ± 3.6
S4				520	0.28	1.32	0.117	0.100	0.144	53.7	33.9 ± 4.3
S5				1000	0.42	1.36	0.077	0.069	0.094	81.6	66.3 ± 4.9

a *M*_sc_, molecular weight of side chains; *n*_sc_, number of side chains per bottlebrush; *n*_end_, DP of end linear PBnMA blocks; *f*, volume fraction of the end blocks; PDI, polydispersity index; q*, wavenumber of the primary scattering peak; d* = 2*π*/q*, characteristic length associated with the primary scattering peak; *D*, domain diameter directly measured from TEM images; and NA, not applicable.

Because our goal is to test interfacial penetration rather than constructing a complete phase diagram, we focus on a small set of representative compositions instead of synthesizing a large library (>20 samples) as in our previous work. We select diblock copolymers with volume fractions *f* = 0.01, 0.08, 0.19, 0.28, and 0.42, spanning a range that captures the major microstructures observed in classical linear block copolymers.^23^ All samples have the same middle bottlebrush block ( $L_{\max}\approx 10\;\mathrm{nm}$ and *b*_*e*_ ≈ 13 nm) but differ only in linear block fraction. This well-defined system isolates architectural effects on self-assembly. Moreover, unlike triblocks, these diblocks contain only one linear end block and therefore cannot form physical networks, providing a clean test of the penetration mechanism.

Following a protocol established in our previous work and others,^24–26^ we anneal all samples in toluene vapor followed by thermal treatment, allowing polymers to rearrange into their equilibrium morphology (Experimental Section, SI). We use transmission electron microscopy (TEM) to visualize the resulting microstructures. The intrinsic contrast between PDMS and PBnMA provides sufficient imaging contrast without staining.^19^ In the processed images, dark regions correspond to PBnMA domains, and bright regions correspond to bottlebrush PDMS domains (Figure 2a). At lower volume fractions (*f* ≤ 0.08), there are no well-defined microstructures in the TEM images due to weak electron density contrast and the absence of long-range order. For *f* ≥ 0.19, all samples exhibit hexagonally packed cylindrical domains, with the PBnMA linear blocks forming the cylindrical cores surrounded by the bottlebrush PDMS matrix. As *f* increases from 0.19 to 0.42, the diameter of the PBnMA cylinders increases from ∼20 nm to ∼70 nm, and the spacing between neighboring cylinders increases correspondingly. The larger domain size and stronger electron density contrast at higher *f* make the hexagonal packing most clearly resolved at *f* = 0.42, as highlighted by the dashed hexagon in Figure 2a­(iii). This is qualitatively consistent with the phase behavior of linear-bottlebrush diblock copolymers, where the conformational asymmetry between the bulky bottlebrush and the linear block shifts the cylinder phase window to higher linear block volume fractions (*f* ≈ 0.3) compared to linear–linear diblocks (*f* ≈ 0.18).^14,15^

**Figure 2:**
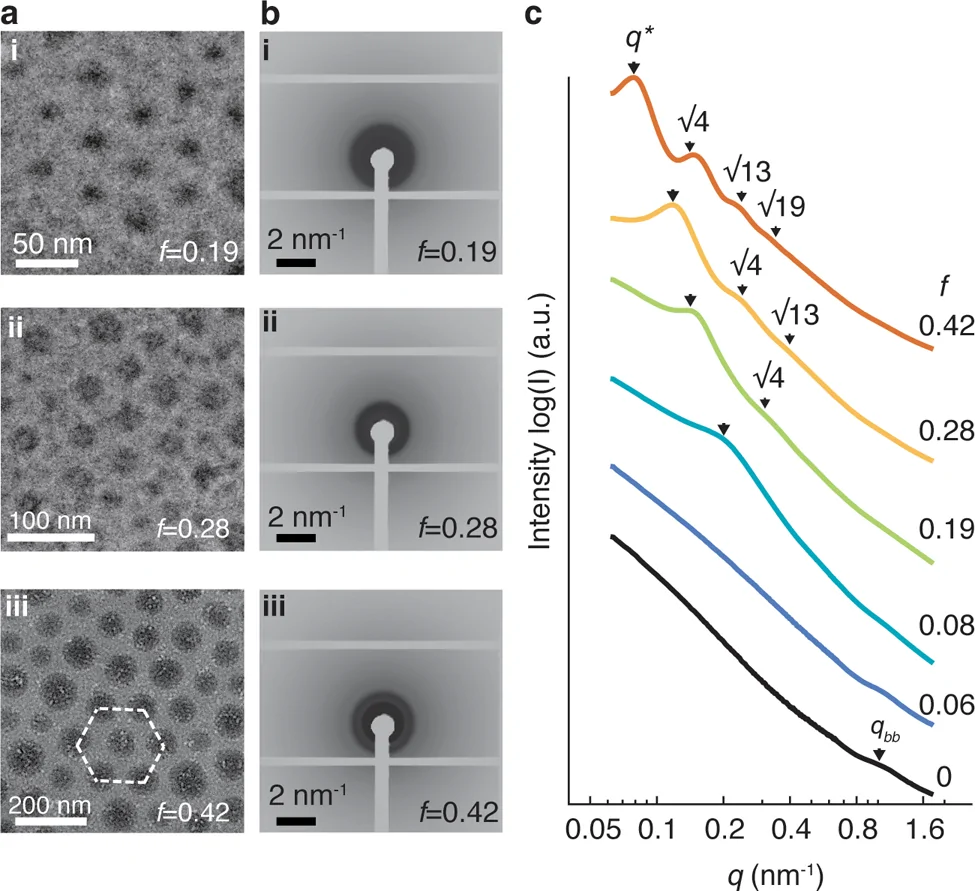
Self-assembled microstructures of LB diblock copolymers. (a) TEM images of LB diblock copolymers with (i) *f* = 0.19, (ii) *f* = 0.28, and (iii) *f* = 0.42. Dark regions correspond to domains formed by linear PBnMA blocks, and bright regions correspond to bottlebrush PDMS domains. The dashed hexagon in (iii) highlights the hexagonal packing of cylindrical microstructures. The contrast is inverted to highlight the self-assembled areas. (b) Corresponding 2D SAXS patterns for samples with (i) *f* = 0.19, (ii) *f* = 0.28, and (iii) *f* = 0.42. Scale bar: 2 nm^–1^. (c) Radially averaged 1D SAXS intensity profiles as a function of the scattering wavevector *q*. The primary scattering peak q* and higher-order peaks at $q/q^{*}\approx \sqrt{4},\sqrt{13},\sqrt{19}$ are consistent with a hexagonal cylinder morphology. The characteristic peak *q*_*bb*_ of pure bottlebrush PDMS (*f* = 0) corresponds to the interbackbone distance (*D*_*bb*_) between neighboring bottlebrush molecules. The associated length scale, *D*_*bb*_ = 2π/*q*_*bb*_ = 6.5 nm, is consistent with our previous measurements.^20^ *D*_*bb*_ is much smaller than twice the size of the side chain, 2*R*_sc_ ≈ 11.6 nm, suggesting interdigitated side chains between neighboring bottlebrush molecules, as illustrated in Figure 3b,c.

To complement TEM measurements, we perform small-/wide-angle X-ray scattering (SAXS/WAXS) using a synchrotron source to characterize the annealed polymers. For each sample, we perform measurements at multiple locations to assess sample uniformity and verify that the scattering features are reproducible. The two-dimensional (2D) SAXS patterns exhibit isotropic rings, indicating randomly oriented but locally ordered microstructures (Figure 2b). After background subtraction and radial averaging, we obtain the one-dimensional (1D) scattering intensity profile as a function of the scattering wavevector *q* (Figure 2c).

We quantify the positions of scattering peaks to identify the microstructure. The primary peak q* is relatively sharp and is readily determined. As *f* increases from 0.08 to 0.42, q* shifts to lower values, indicating an increase in characteristic length scales, consistent with the TEM observations. By contrast, higher-order peaks are broad, making their positions less well-defined. This broadening reflects limited long-range order in the self-assembled structure. A diffuse interfacial boundary in block copolymers would not alter peak location but would further suppress the intensity of higher-order peaks relative to the primary peak, making them difficult to locate precisely.^27^ To this end, we apply the derivative method established in our previous work to determine the lower and upper bounds of each peak (Figure S1).

For *f* ≥ 0.19, the peak ratios, $q/q^{*}\approx \sqrt{1},\sqrt{4},\sqrt{13},\sqrt{19}$ , indicate hexagonal cylinder morphology. Notably, no transitions to the lamellar phase are observed even at *f* = 0.42, contrasting with semiflexible-linear block copolymers.^28^ This behavior is consistent with our prior results on LBL triblock copolymers,^20^ which also remain cylindrical morphology up to *f* = 0.40. Determining the upper composition limit for the cylinder phase would be of interest; however, accessing higher *f* in LB polymers requires very high MW linear blocks (degree of polymerization >1000), which represents a challenge for atom transfer radical polymerization (ATRP) and will be explored in future work. Nevertheless, combined TEM and SAXS results confirm that all samples with 0.19 ≤ *f* ≤ 0.42 exhibit a hexagonal cylinder morphology (Figure 3a).

**Figure 3:**
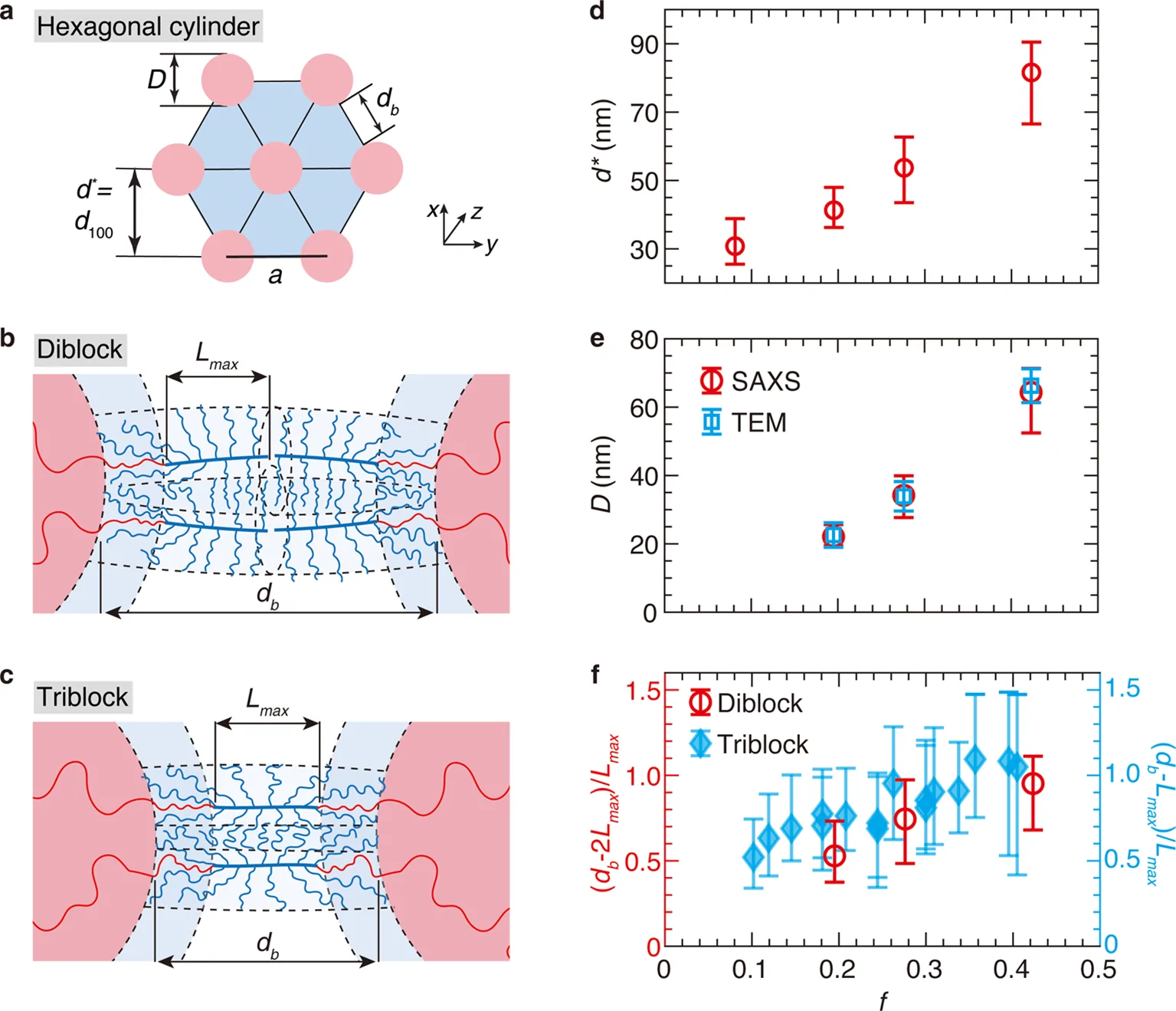
Large domain spacing originates from the penetration of linear tails into the bottlebrush domain. (a) Schematic of a hexagonal cylinder microstructure in the *x-y* plane, perpendicular to the cylinder axis (*z*), where *D* is the diameter of the linear block domain, *d*_*b*_ is the bridging distance between neighboring linear domains, d* = *d*_100_ is the characteristic length corresponding to the primary scattering peak, and *a* is the lattice constant $a=\left(2/\sqrt{3}\right)d^{*}$ . (b, c) Molecular picture of the self-assembled diblock copolymer and triblock copolymers in the same *x–y* plane as (a). The end linear blocks (red lines) are pulled out to generate additional space for the long side chains, alleviating packing frustration near curved interfaces and effectively increasing the size of the bottlebrush domain. (d) Dependence of the primary characteristic length d* on the volume fraction *f* of linear blocks. (e) Dependence of the linear block domain diameter *D* on *f*. Red circles: data calculated from SAXS measurements; blue squares: data directly measured from TEM images. (f) Normalized bridging distance as a function of *f*. Red circles (left axis): diblock copolymers; blue diamonds (right axis): triblock copolymers from our previous work.^20^ The overlap of the two data sets confirms that linear chains penetrate the bottlebrush domain from one end in diblocks and from both ends in triblocks. The error bars in (d)–(f) represent the peak widths extracted from SAXS measurements using the derivative method (Figure S1).

For hexagonally packed cylinders, the characteristic length d* = 2π/q* corresponds to the first Bragg reflection *d*_100_, and the lattice constant is $a=\left(2/\sqrt{3}\right)d^{*}$ ­(Figure 3a). The average diameter of the PBnMA cylinders is $D={\left(6/\sqrt{3}\pi \right)}^{1/2}f^{1/2}a$ , derived from conservation of the linear block volume within the hexagonal unit cell. The domain spacing, or the bridging distance between two neighboring cylinders, is *d*_*b*_ = *a* – *D*. As *f* increases from 0.19 to 0.42, d* increases from ∼30 nm to ∼80 nm (Figure 3d). The values of *D* calculated from SAXS (red circles in Figure 3e) agree well with those directly measured from TEM (blue squares in Figure 3e, Figure S2), supporting the reliability of the calculated *D* and *d*_*b*_.

We compare the bridging distance *d*_*b*_ with the maximum span set by the bottlebrush backbone. For an LB diblock bridge between two neighboring PBnMA cylinders, the bottlebrush domain contains two opposing molecules, each contributing one penetrated linear end (Figure 3b). We therefore define the excess bridging distance as ( $d_{b}-2L_{\max})/L_{\max}$ (Figure 3f). This quantity exceeds zero across the explored volume fractions, confirming that *d*_*b*_ surpasses the bottlebrush backbone contour length. Importantly, these values are in quantitative agreement with the excess distance in triblocks, ( $d_{b}-L_{\max})/L_{\max}$ , where the bottlebrush domain contains only one bottlebrush block (diamonds, Figure 3f). Moreover, the absolute values of the excess lengths, $d_{b}-2L_{\max}$ for diblocks and $d_{b}-L_{\max}$ for triblocks, are approximately 10 nm, much larger than the interfacial thickness (∼1 nm) in strongly segregated linear block copolymers. Together, these results demonstrate that domain spacing in architecturally asymmetric (LB or LBL) block copolymers can significantly exceed the bottlebrush contour length.

Note that incomplete macroinitiator activation could generate unreacted bottlebrush PDMS. These macromolecules, however, are unlikely to cause anomalously large domain spacing. Fractional precipitation preferentially removes low-molecular-weight bottlebrushes, particularly for samples with long PBnMA blocks, in which excess bridging distance persists. Moreover, non-network-forming species may increase domain spacing but cannot explain bridging distances beyond the bottlebrush contour length in linear–bottlebrush–linear triblocks.

To rationalize the anomalously large domain spacing, we propose a mechanism based on interfacial chain penetration. Interfacial repulsion between the incompatible linear and bottlebrush domains generates tension along the polymer backbone, pulling segments of the linear block into the bottlebrush domain. This penetration increases the bridging distance and creates additional space for densely grafted side chains near the bottlebrush ends, thereby relieving the packing frustration and reducing their stretching entropic penalty. Although penetration incurs an interfacial penalty and requires stretching of the invaded linear segment, a scaling analysis indicates that the resulting gains in side chain entropy and the reduction in domain interfacial area outweigh these costs (Figure S3 and SI Text). Consequently, interfacial chain penetration is energetically favorable and provides a physical mechanism for domain spacing that exceeds the bottlebrush contour length.

We do not envision complete mixing of the PBnMA linear block within the PDMS bottlebrush domain. Instead, a limited portion of the linear chain invades the bottlebrush domain. Because the required penetration length is small relative to the contour length of the linear chains, this mechanism can produce substantial increases in domain spacing. Consistent with this picture, our earlier experimental work^19^ *serendipitously* showed that the excess length increases with side-chain length (i.e., bottlebrush bulkiness), which we now attribute to enhanced interfacial penetration. The proposed mechanism further leads to testable predictions. Lowering the temperature should increase χ and the interfacial penalty associated with linear chain penetration, thereby decreasing the excess bridging distance. Conversely, selectively swelling the PDMS bottlebrush domain should increase side chain size, enhance steric repulsion, and further promote linear chain penetration.

Beyond explaining the anomalously large domain spacing observed in LB and LBL block copolymers, interfacial chain penetration suggests a broader mechanism by which the molecular architecture can reshape interfacial structure and self-assembly pathways. In strongly segregated linear block copolymers, interfaces are typically treated as narrow boundaries governed primarily by the segment length and incompatibility. In architecturally asymmetric copolymers, interfacial chain penetration may provide a general mechanism for relieving packing frustration and increasing characteristic domain spacing beyond the limit expected from the chain contour length. This mechanism may also shift phase boundaries and stabilize curved morphologies over a broad composition range. Indeed, the experimentally determined composition ranges for different phases^20^ are broader than those predicted by self-consistent field theory.^18^ More broadly, our findings suggest that architectural asymmetry serves as a design parameter for programming the domain spacing and morphology in nanostructured soft materials.

## Supplementary Material



## Data Availability

All data and code needed to evaluate and reproduce the results in the paper are present in the paper and/or the Supporting Information. Materials generated in this study may be available from the corresponding author upon reasonable request. Requests for materials should be directed to LHC (liheng.cai@virginia.edu).

## Notes

Materials and Methods; Additional characterization by SAXS and TEM; Supporting text, including theoretical calculations and discussions; ^1^H NMR spectra for all samples (PDF)

## References

[1] Lodge T. P. (2003). Block Copolymers:
Past Successes and Future Challenges. Macromol.
Chem. Phys..

[2] Bates C. M., Bates F. S. (2017). 50th Anniversary Perspective: Block Polymers-Pure Potential. Macromolecules.

[3] Segalman R. A. (2005). Patterning
with Block Copolymer Thin Films. Mater. Sci.
Eng., R.

[4] Hillmyer, M. A. Nanoporous Materials from Block Copolymer Precursors. Block Copolymers II; Springer-Verlag: Berlin, Heidelberg, 2005; pp 137–181. 10.1007/12_002.

[5] Kataoka K., Harada A., Nagasaki Y. (2012). Block Copolymer
Micelles for Drug
Delivery: Design, Characterization and Biological Significance. Adv. Drug Delivery Rev..

[6] Bates F. S., Fredrickson G. H. (1990). Block Copolymer Thermodynamics: Theory
and Experiment. Annu. Rev. Phys. Chem..

[7] Helfand E., Tagami Y. (1972). Theory of the Interface between Immiscible Polymers.
II. J. Chem. Phys..

[8] Helfand E., Sapse A. M. (1975). Theory of Unsymmetric
Polymer-Polymer Interfaces. J. Chem. Phys..

[9] Paturej J., Sheiko S. S., Panyukov S., Rubinstein M. (2016). Molecular
Structure of Bottlebrush Polymers in Melts. Sci. Adv..

[10] Li Y., Zou J., Das B. P., Tsianou M., Cheng C. (2012). Well-Defined Amphiphilic
Double-Brush Copolymers and Their Performance as Emulsion Surfactants. Macromolecules.

[11] Fenyves R., Schmutz M., Horner I. J., Bright F. V., Rzayev J. (2014). Aqueous Self-Assembly
of Giant Bottlebrush Block Copolymer Surfactants as Shape-Tunable
Building Blocks. J. Am. Chem. Soc..

[12] Kerai S. D., Takano S., Zeng P., Müllner M. (2025). Self-Assembly
of Bottlebrush-Linear, Rod-Coil Copolymers into Discoidal Nanoparticles. ACS Macro Lett..

[13] Zhang J., Song Q., Peng L., Huang X., Li W. (2025). Square Array
of Cylinders Formed in Linear-Bottlebrush-Linear Triblock Copolymers. Macromolecules.

[14] Liberman L., Coughlin M. L., Weigand S., Edmund J., Bates F. S., Lodge T. P. (2022). Impact of Side-Chain
Length on the Self-Assembly of
Linear-Bottlebrush Diblock Copolymers. Macromolecules.

[15] Liberman L., Coughlin M. L., Weigand S., Bates F. S., Lodge T. P. (2022). Phase Behavior
of Linear-Bottlebrush Block Polymers. Macromolecules.

[16] Kang H., Vo J. N., Huan X., Diao Y., Guironnet D., Sing C. E. (2026). Implicit Side-Chain Model for Melt Bottlebrush Block
Copolymer Assembly. Macromolecules.

[17] Park J., Nam J., Seo M., Li S. (2022). Side-Chain Density Driven Morphology
Transition in Brush-Linear Diblock Copolymers. ACS Macro Lett..

[18] Zhulina E. B., Sheiko S. S., Dobrynin A. V., Borisov O. V. (2020). Microphase
Segregation
in the Melts of Bottlebrush Block Copolymers. Macromolecules.

[19] Nian S., Lian H., Gong Z., Zhernenkov M., Qin J., Cai L.-H. (2019). Molecular Architecture
Directs Linear-Bottlebrush-Linear
Triblock Copolymers to Self-Assemble to Soft Reprocessable Elastomers. ACS Macro Lett..

[20] Nian S., Fan Z., Freychet G., Zhernenkov M., Redemann S., Cai L.-H. (2021). Self-Assembly
of Flexible Linear-Semiflexible Bottlebrush-Flexible Linear Triblock
Copolymers. Macromolecules.

[21] Nian S., Zhu J., Zhang H., Gong Z., Freychet G., Zhernenkov M., Xu B., Cai L.-H. (2021). Three-Dimensional Printable, Extremely Soft, Stretchable,
and Reversible Elastomers from Molecular Architecture-Directed Assembly. Chem. Mater..

[22] Huang B., Nian S., Cai L.-H. (2024). A Universal
Strategy for Decoupling
Stiffness and Extensibility of Polymer Networks. Sci. Adv..

[23] Matsen M. W., Bates F. S. (1996). Unifying Weak- and Strong-Segregation Block Copolymer
Theories. Macromolecules.

[24] Kim S. H., Misner M. J., Xu T., Kimura M., Russell T. P. (2004). Highly
Oriented and Ordered Arrays from Block Copolymers via Solvent Evaporation. Adv. Mater..

[25] Gotrik K. W., Ross C. A. (2013). Solvothermal Annealing of Block Copolymer Thin Films. Nano Lett..

[26] Sinturel C., Vayer M., Morris M., Hillmyer M. A. (2013). Solvent Vapor Annealing
of Block Polymer Thin Films. Macromolecules.

[27] Koberstein J. T., Morra B., Stein R. S. (1980). The Determination of Diffuse-Boundary
Thicknesses of Polymers by Small-Angle X-Ray Scattering. J. Appl. Crystallogr..

[28] Koga M., Abe K., Sato K., Koki J., Kang S., Sakajiri K., Watanabe J., Tokita M. (2014). Self-Assembly of Flexible-Semiflexible-Flexible
Triblock Copolymers. Macromolecules.

