# Supplementary material for: Interfacial Penetration Drives Anomalous Domain Spacing in Strongly Segregated Linear-Bottlebrush Copolymers
Source: ACS Macro Lett. 2026 Aug 20;15(9):1222–7. doi: 10.1021/acsmacrolett.6c00296 (PMC13585141; doi:10.1021/acsmacrolett.6c00296)
Supplement: Supplementary file 1 [file mz6c00296_si_001.pdf]

Supporting Information

Interfacial penetration drives anomalous domain spacing in strongly segregated linear-bottlebrush copolymers

Baiqiang Huang[1], Haobo Wang[1], Patryk Wąsik[2], Eliot Gann[2], and Li-Heng Cai[1,3,4,5*]

Affiliations:

[1]Soft Biomatter Laboratory, Department of Materials Science and Engineering, University of Virginia, Charlottesville, VA 22904, USA
[2]National Synchrotron Light Source-II, Brookhaven National Laboratory, Upton, NY 11973, USA
[3]Department of Chemical Engineering, University of Virginia, Charlottesville, VA 22904, USA
[4]Department of Biomedical Engineering, University of Virginia, Charlottesville, VA 22904, USA
[5]Department of Chemistry, University of Virginia, Charlottesville, VA 22904, USA

*Corresponding author. Li-Heng Cai: liheng.cai@virginia.edu

Supporting Information:

SI Text
SI Materials and Methods
Figures S1-S10
References

SI Text

Free-energy argument for interfacial chain penetration

We estimate the free energy change associated with interfacial chain penetration. Without penetration, side chains near the domain boundary remain stretched (**Fig. S3a**). As the linear block penetrates the bottlebrush domain, it adds additional interfacial free energy, ΔF_{int}^{L}, between the "invaded" side chain and the bottlebrush side chains. This process also increases the bridging distance d_b. To maintain the volume fraction of the linear block, the size of the linear block domain must increase. Consequently, the total interfacial area decreases, resulting in a reduction of the interfacial free energy, ΔF_{int}^{BB}, between the linear and bottlebrush domains.

Interfacial chain penetration also creates additional space for side chains near the bottlebrush end, reducing side chain crowding and stretching and thereby producing an entropic free energy gain, ΔF_{ent}^{sc} (**Fig. S3b**). At the same time, stretching of the penetrated linear block incurs an entropic penalty, ΔF_{ent}^{L}. The question therefore is whether the total free energy change ΔF_{tot} favors interfacial chain penetration.

$$\Delta F_{tot} = \Delta F_{int}^{L} + \Delta F_{int}^{BB} + \Delta F_{ent}^{L} + \Delta F_{ent}^{sc} \quad \text{(S1)}$$

1. Entropic free energy change associated with side chain stretching

We start by estimating the entropic free energy change ΔF_{ent}^{sc} associated with side chain stretching. In a densely grafted bottlebrush polymer, the grafting density, $1/N_g$, is no less than a crossover value, $1/N_g^{**}$, at which the backbone section between two neighboring grafting sites is stretched to its maximum length:

$$N_g^{**} \approx \frac{v_{sc}}{b_{sc} l_{sc} l_{bb}} \quad \text{(S2)}$$

where l, v, b, respectively, denote the length of all main-chain bonds of a chemical monomer, the volume of a chemical monomer, and the length of a Kuhn segment. We use '*sc*' and '*bb*' as subscripts or superscripts to denote side chains and backbone, respectively.

In our bottlebrush polymers, the side chains are linear PDMS, and the backbone is poly(methyl methacrylate) (PMMA): $b_{sc} = 13.0$ Å, $l_{sc} = 2.55$ Å, $v_{sc} = 127$ Å^3, and $l_{bb} = 2.56$ Å. These

give $N_g^{**} \approx 1.5$, which is greater than $N_g = 1$ in our design, suggesting our design is a densely grafted bottlebrush polymer[1].

In this regime, the side chain R_{sc} is determined by volume conservation: $R_{sc}^2 N_g l_{bb} \approx v_{sc} N_{sc}$, where $N_{sc} \approx 68$ is the degree of polymerization of the side chain. This gives:

$$R_{sc} \approx \left(\frac{v_{sc} N_{sc}}{N_g l_{bb}}\right)^{\frac{1}{2}} \approx R_{sc,0} \left(\frac{N_g^{**}}{N_g}\right)^{1/2} \tag{S3}$$

where $R_{sc,0} \approx (b_{sc} l_{sc} N_{sc})^{\frac{1}{2}} \approx 4.7$ nm is the unperturbed size of the side chain, where $b_{sc} = 1.3$ nm is the Kuhn length of PDMS. Thus, for each side chain, the entropic free energy due to stretching is

$$F_{ent}^{sc,0} \approx k_B T \left(\frac{R_{sc}}{R_{sc,0}}\right)^2 \approx k_B T \frac{N_g^{**}}{N_g} \tag{S4}$$

When the linear block penetrates the bottlebrush domain, the side chains can occupy the space at the bottlebrush end to reach an unperturbed size. The number of side chains that can do so is approximately $\delta l / l_{bb}$. Thus, the entropic free energy change associated with side chain stretching is

$$\Delta F_{ent}^{sc} \approx -k_B T \left(\frac{N_g^{**}}{N_g} - 1\right) \left(\frac{\delta l}{l_{bb}}\right) \tag{S5}$$

2. Entropic penalty associated with stretching the penetrated linear segment

To calculate the entropic penalty of stretching the invaded linear segment, we consider the force or tension f_T along the invaded chain, which is comparable to that of stretching the bottlebrush backbone. The bottlebrush backbone size follows[2]

$$R_{bb} \approx R_{bb,0} \left(\frac{R_{sc,0}}{b_{bb}}\right)^{\frac{1}{2}} \left(\frac{N_g^{**}}{N_g}\right)^{\frac{1}{2}} \tag{S6}$$

The entropic free energy associated with the stretched bottlebrush backbone is

$$F_{ent}^{bb} \approx k_B T \left(\frac{R_{bb}}{R_{bb,0}}\right)^2 \approx k_B T \left(\frac{R_{sc,0}}{b_{bb}}\right)\left(\frac{N_g^{**}}{N_g}\right) \tag{S7}$$

At the scaling level, the tension along the bottlebrush backbone is $f_T \approx F_{ent}^{bb}/\mathcal{L}_{\max}$, where $\mathcal{L}_{\max}$ is the bottlebrush backbone contour length. Assuming that a comparable tension stretches the penetrated linear segment gives:

$$\Delta F_{ent}^{L} \approx F_{ent}^{bb} \frac{\delta l}{\mathcal{L}_{\max}} \approx k_B T \left(\frac{R_{sc,0}}{b_{bb}}\right)\left(\frac{N_g^{**}}{N_g}\right)\frac{\delta l}{\mathcal{L}_{\max}} \tag{S8}$$

3. Interfacial penalty associated with linear chain penetration

The penetrated linear segment creates unfavorable contacts with the PDMS side chains. A scaling estimate for this interfacial penalty is:

$$\Delta F_{int}^{L} \approx k_B T \chi \left(\frac{\delta l}{b}\right) \tag{S9}$$

Here, $\chi \approx 0.2$ is the Flory-Huggins interaction parameter between PBnMA and PDMS, and $b = 18.6$ Å is the Kuhn length of linear PBnMA.

4. Reduction in domain interfacial free energy

Finally, let's consider the reduction of interfacial free energy, ΔF_{int}^{BB}, between the linear and bottlebrush domains due to interfacial chain penetration. We use cylindrical morphology as an example to estimate the contribution per block copolymer.

The interfacial free energy per diblock copolymer:

$$F_{int}^{BB} \approx \gamma A/n \tag{S10}$$

For a unit cell with cylinder length of L, the total domain interfacial area $A = 3\pi DL$, and the number of block copolymers $n = \left(\frac{3\pi}{4}\right) D^2 L/[Mf/(N_A\rho)]$, where M is the molar mass of the

copolymer, f is the volume fraction of the linear block, N_A is the Avogadro number, and ρ is the polymer density. Thus,

$$F_{int}^{BB} \approx \frac{4\gamma M f}{\rho N_A}\frac{1}{D} \tag{S11}$$

The interfacial tension scales as $\gamma \approx k_B T b^{-2}\chi^{1/2}$. For hexagonally packed cylinders, the cylinder diameter is $D = (6/\sqrt{3}\pi)^{1/2} f^{1/2} a \approx f^{1/2}(D + d_b)$, where $a = D + d_b$; this gives $D \approx d_b/(f^{-1/2} - 1)$. Substituting this relation into eq. (S11) gives:

$$F_{int}^{BB} \approx k_B T \frac{4M}{N_A \rho b^2}\chi^{\frac{1}{2}}\left(f^{\frac{1}{2}} - f\right)\frac{1}{d_b} \tag{S12}$$

Without penetration, $d_b = 2\mathcal{L}_{\max}$; whereas with linear chain penetration, $d_b = 2\mathcal{L}_{\max} + 2\delta l$. Defining ΔF_{int}^{BB} as the free energy of the penetrated state relative to that of the nonpenetrated state, the interfacial free-energy change per diblock copolymer is:

$$\Delta F_{int}^{BB} \approx -k_B T \frac{2M}{N_A \rho b^2}\chi^{\frac{1}{2}}\left(f^{\frac{1}{2}} - f\right)\frac{\delta l}{\mathcal{L}_{\max}(\mathcal{L}_{\max} + \delta l)} \tag{S13}$$

The negative sign indicates that interfacial chain penetration reduces the interfacial free energy.

Taken together, the total free energy change ΔF_{tot} associated with interfacial chain penetration of depth δl is:

$$\frac{\Delta F_{tot}}{k_B T} \approx -\left(\frac{N_g^{**}}{N_g} - 1\right)\left(\frac{\delta l}{l_{bb}}\right) + \chi\left(\frac{\delta l}{b}\right) + \left(\frac{R_{sc,0}}{b_{bb}}\right)\left(\frac{N_g^{**}}{N_g}\right)\frac{\delta l}{\mathcal{L}_{\max}} + \frac{\Delta F_{int}^{BB}}{k_B T} \tag{S14}$$

For the sample with high volume fraction (Sample S5) that exhibits relatively large penetration depth $\delta l \approx 5$ nm, $N_g = 1$, $N_g^{**} \approx 1.5$, $l_{bb} = 2.56$ Å, $b = 18.6$ Å, $\mathcal{L}_{\max} \approx 10$ nm, $R_{sc,0} \approx 4.7$ nm, and $b_{bb} = 1.7$nm. The first three contributions give:

$$\Delta F_{int}^{L} + \Delta F_{ent}^{L} + \Delta F_{ent}^{sc} \approx -7k_B T \tag{S15}$$

Because $\Delta F_{int}^{BB} < 0$, including the reduction in domain interfacial free energy makes ΔF_{tot} even more negative. Thus, this scaling analysis suggests that interfacial chain penetration is energetically favorable in asymmetric linear–bottlebrush and linear–bottlebrush–linear block copolymers. However, an analytical theory and simulations are still needed to quantitatively determine how molecular architecture affects interfacial structure and self-assembly pathways.

SI Materials and Methods

Materials

Monomethacryloxypropyl-terminated PDMS (MCR-M17, 5 kg/mol, Gelest) and benzyl methacrylate (BnMA, 96%, Sigma-Aldrich) are purified using basic aluminum oxide columns to remove inhibitors before use. Copper(II) chloride ($CuCl_2$, 99.999%), tris[2-(dimethylamino)ethyl]amine (Me_6TREN), ethyl 2-bromobutyrate (EBriB, 99%), tin(II) 2-ethylhexanoate [$Sn(EH)_2$, 92.5−100%], anisole (≥99.7%), and p-xylene (≥99.7%) are purchased from Sigma-Aldrich and used as received. Methanol (Certified ACS), diethyl ether (Certified ACS), dimethylformamide (DMF, Certified ACS), tetrahydrofuran (THF, Certified ACS), and THF (HPLC) are purchased from Fisher and used as received.

Synthesis and characterization of linear-bottlebrush (LB) diblock copolymers

To synthesize an LB diblock copolymer, we first synthesize the bottlebrush block and then use it as a macroinitiator to grow the linear block. Here, we describe the detailed synthesis protocol, using the sample with the highest volume fraction as an example.

Step I. Synthesis of bottlebrush polymer. We synthesize a bottlebrush PDMS polymer using ARGET-ATRP[20-24]. In a 50 mL Schlenk flask, we add the initiator EBriB (4.9 mg, 0.025 mmol), MCR-M17 (20.0 g, 4.0 mmol), *p*-xylene (6 mL), and anisole (6 mL). Separately, we prepare the catalyst solution by dissolving Me_6TREN (17.3 mg) and $CuCl_2$ (1 mg) in 1 mL of DMF. After adding 100 μL of catalyst solution to the flask, we bubble the mixture with nitrogen for 60 min. Then, we inject the reducing agent $Sn(EH)_2$ (12 mg, dissolved in 200 μL of p-xylene) and seal the flask. The reaction proceeds at 60 °C in an oil bath and is quenched in liquid nitrogen at 25% conversion, yielding bottlebrush PDMS with a degree of polymerization (DP) of 40, as confirmed by ^{1}H NMR (**Fig. S3**). We dilute the reaction mixture with THF and pass it through a neutral alumina column to remove the catalyst. The solution is then concentrated using a rotary evaporator. To separate the bottlebrush polymer from unreacted macromonomers, we use a co-solvent of methanol and diethyl ether (3:2 by volume), which dissolves the macromonomers but not the bottlebrush PDMS. After five precipitation procedures, the unreacted macromonomers are completely removed. GPC confirms a PDI of 1.20 for the purified bottlebrush PDMS (**Fig. 1c**).

Step II. Synthesis of LB diblock copolymers. We use the purified bottlebrush as a macroinitiator to grow the linear PBnMA block using ARGET-ATRP. In a 25 mL Schlenk flask, we combine BnMA (2.6 g, 15 mmol), bottlebrush PDMS macroinitiator (600 mg, 3×10^{-3} mmol), *p*-xylene (6 mL), and anisole (6 mL). The catalyst solution and reaction procedure are the same as in *Step I*. After quenching in liquid nitrogen, we dilute the mixture with THF and pass it through a neutral alumina column. For purification, we use a co-solvent of methanol and acetone (1:1 by volume), which removes unreacted monomers and free PBnMA chains. After three precipitation cycles, the polymer is dried under vacuum at room temperature for 24 h. The degree of polymerization of the PBnMA block is 1000, and the PDI of the LB diblock copolymer is 1.36 (**Fig. S9**, **Table 1**).

<u>^{1}H NMR characterization.</u> Proton nuclear magnetic resonance (^{1}H NMR) spectroscopy is performed using a Varian NMRS 600 MHz spectrometer. For all samples, deuterated chloroform ($CDCl_3$) is used as a solvent.

<u>GPC characterization.</u> GPC measurements are performed using TOSOH EcoSEC HLC-8320 GPC system with two TOSOH Bioscience TSKgel GMHHR-M 5µm columns in series and a refractive index detector at 40°C. THF (HPLC grade) is used as the eluent with a flow rate of 1 mL/min. The samples are dissolved in THF with a concentration of around 5 mg/mL.

<u>Transmission electron microscopy.</u> We prepare samples for TEM imaging using a combination of solvent vapor annealing and thermal treatment. The polymer is dissolved in toluene at a concentration of 5 mg/mL and filtered through a 0.45 µm syringe filter. We deposit 10 µL of the solution onto a carbon film-coated copper TEM grid placed inside a sealed glass chamber with toluene at the bottom. The chamber is then transferred to an oven at 100 °C for 24 h, generating a toluene vapor pressure of approximately 75 kPa. Afterward, the oven is cooled to room temperature slowly.

TEM measurement is performed using a Thermo Fisher Scientific Themis Z microscope operated at an accelerating voltage of 200 keV in conventional bright-field mode to characterize the annealed samples. To enhance contrast from the self-assembled polymer domains, images are acquired under intentionally defocused conditions. The characteristic size of the cylindrical

domains is quantified using ImageJ software, and more than 200 domains with independent image regions are analyzed to ensure statistically meaningful results.

SAXS/WAXS measurements. For SAXS/WAXS measurements, we dissolve the polymer in toluene at a concentration of 100 mg/mL and load the solution into thin-walled quartz capillary tubes (1.5 mm diameter). Then, the capillaries are annealed at 180 °C in a vacuum oven for 24 h, then slowly cooled to room temperature and further annealed for 3 days.

SAXS/WAXS measurements are performed at the Soft Matter Interfaces (12-ID) beamline at the National Synchrotron Light Source II, Brookhaven National Laboratory. We use an X-ray wavelength of $\lambda = 0.77$ Å (16.1 keV) and sample-to-detector distances of 8.3 m for SAXS and 0.28 m for WAXS. Scattered X-rays are collected using an in-vacuum Pilatus 2M (SAXS) and Pilatus 900k-W (WAXS) detectors. The two-dimensional scattering patterns are radially integrated using a custom Python code to obtain one-dimensional intensity profiles *I(q)* as a function of the scattering wavevector $q = 4\pi\lambda^{-1} sin(\theta/2)$, where θ is the scattering angle.

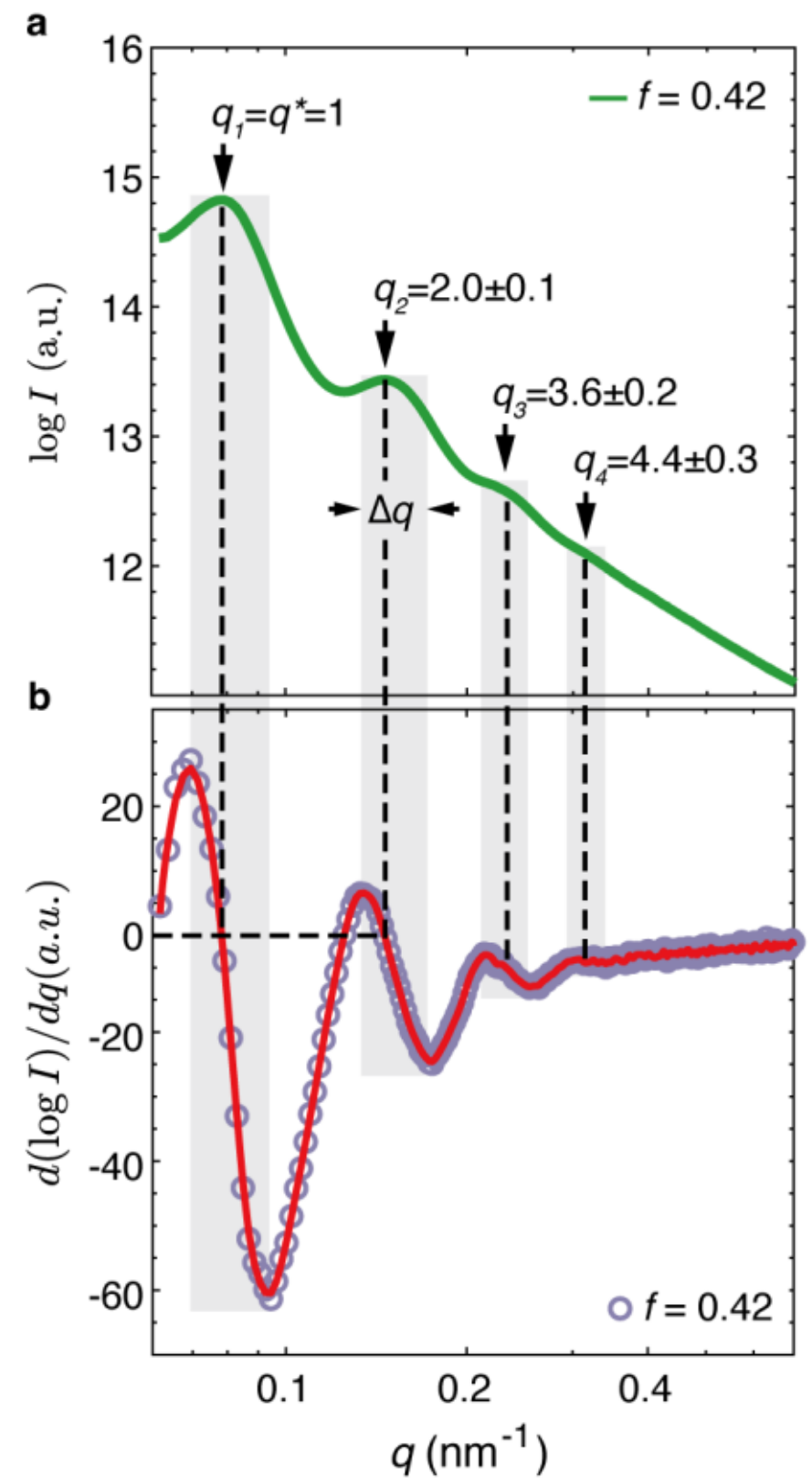

Fig. S1. Determination of peak positions in the SAXS profile of sample S5 ($f = 0.42$) using the derivative method. **(a)** Radially averaged 1D scattering intensity I as a function of wavevector q. The primary peak is at $q^* = 1$, and higher-order peaks are identified at $q_2 = 2.0 \pm 0.1$, $q_3 = 3.6 \pm 0.2$, and $q_4 = 4.4 \pm 0.3$ (in units of q^*). **(b)** Derivative d(log I)/dq as a function of q. The solid red line is a spline fit to the data. The gray regions indicate the width of each peak, defined by the local maximum and minimum of the derivative, and are used as error bars in **Fig. 3**.

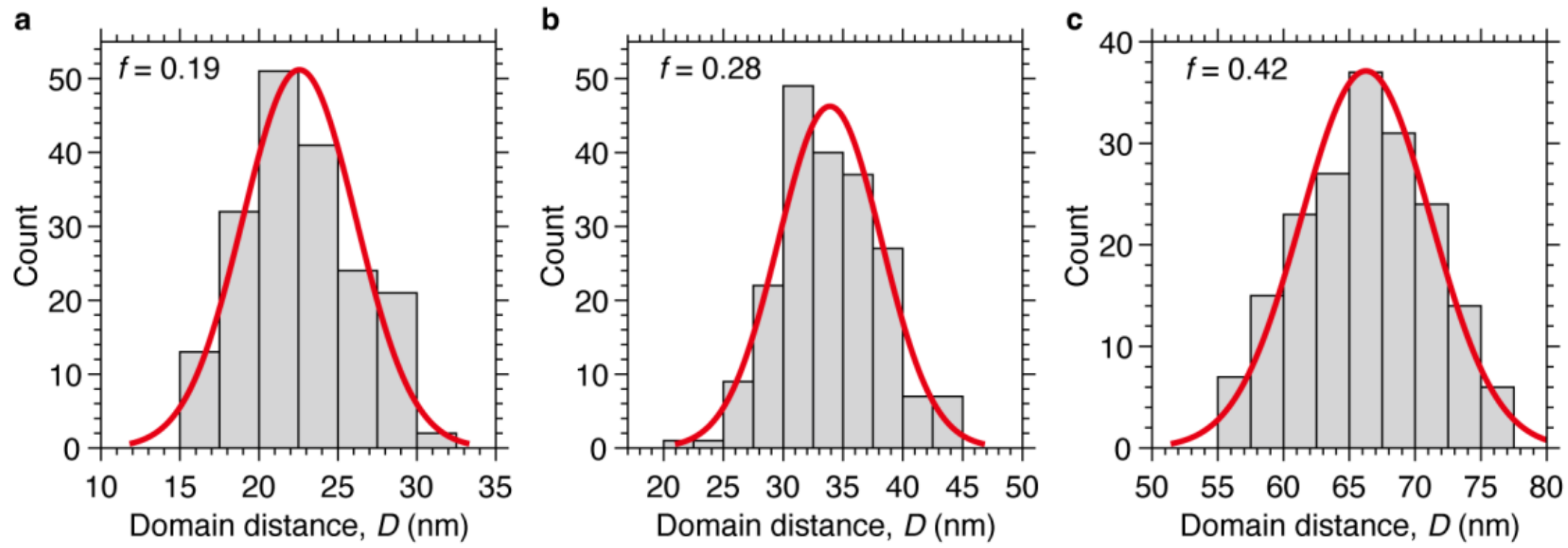

Fig. S2. Domain diameter D measured directly from TEM images for samples with **(a)** $f = 0.19$, **(b)** $f = 0.28$, and **(c)** $f = 0.42$. The histograms are fitted with Gaussian distributions (red curves), and the mean and standard deviation are used as the reported values in **Table 1** and error bars in **Fig. 3e**.

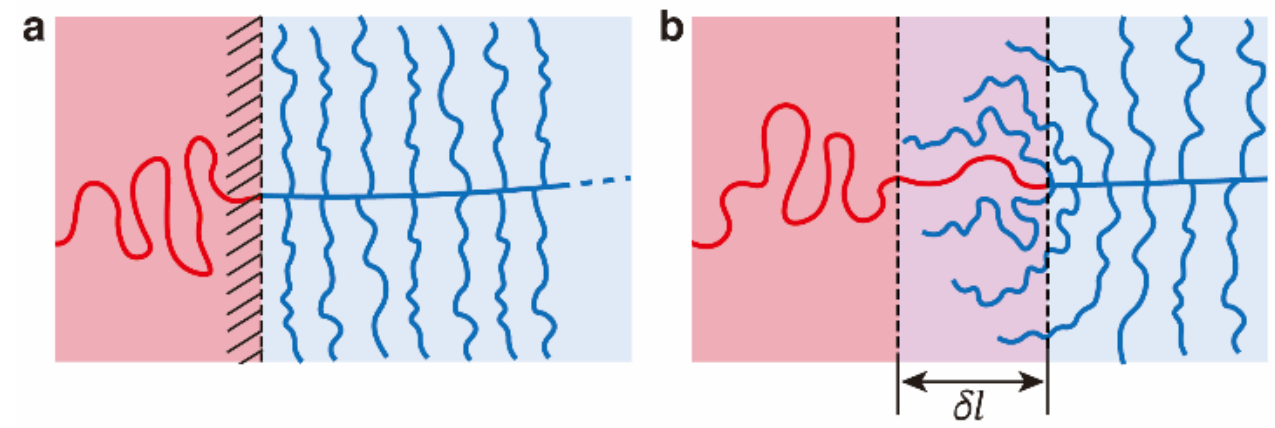

Fig. S3. Side-chain relaxation upon interfacial chain penetration. Schematic cross section of the domain boundary between the PBnMA linear domain (red) and the PDMS bottlebrush domain (blue), in the *y*-*z* plane of **Fig. 3a**. **(a)** Without penetration, side chains near the domain boundary remain stretched. **(b)** As the linear block penetrates the bottlebrush domain, it creates additional interfacial free energy between the "invaded" linear chain and the bottlebrush side chains. Simultaneously, this process provides more space for side chains near the bottlebrush end, such that these side chains are not crowded and thus less stretched, resulting in entropic free energy gain. For linear-bottlebrush polymers with high volume, the penetration depth δl is about 5 nm.

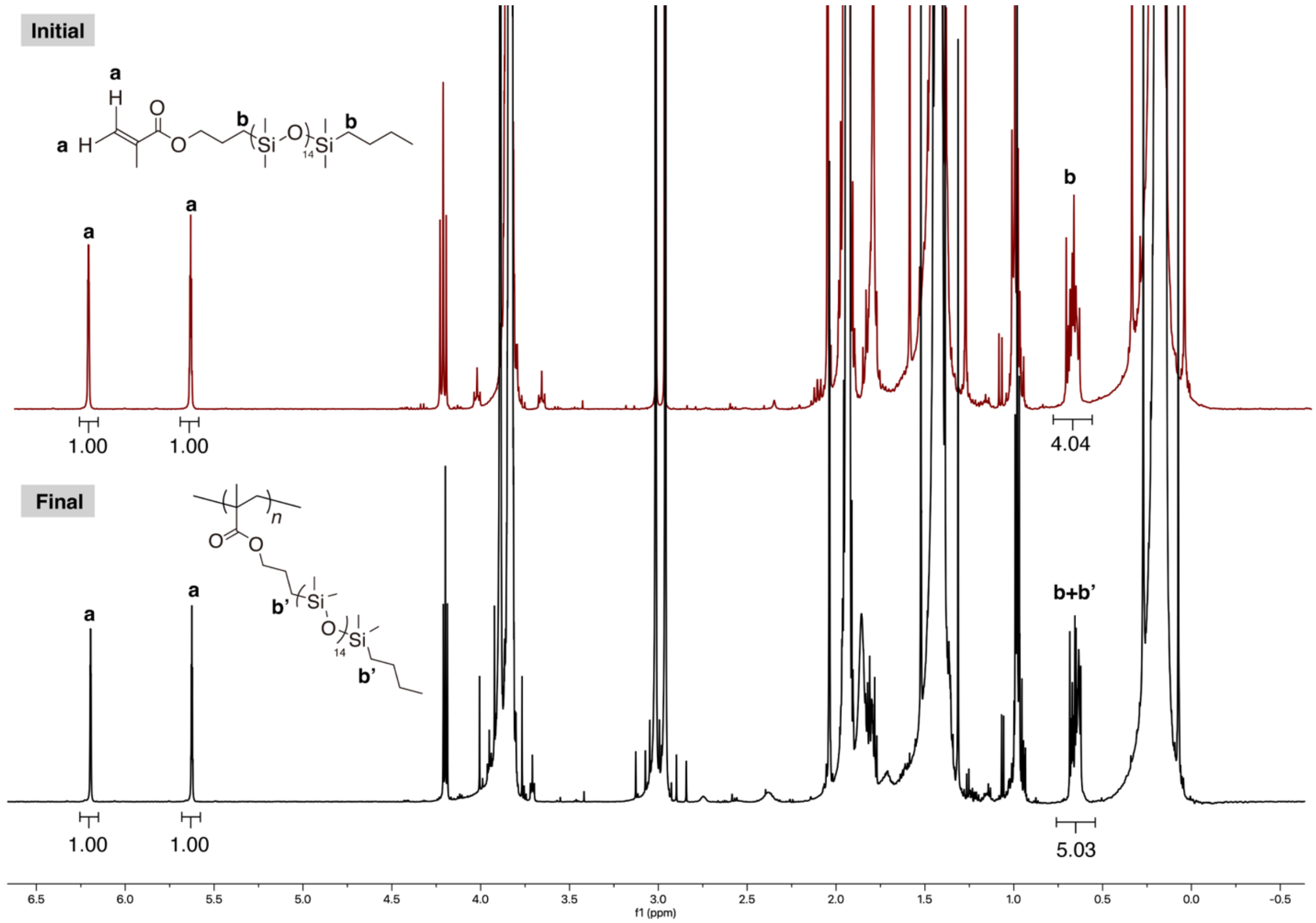

Fig. S4. ^{1}H NMR spectra of the PDMS bottlebrush reaction mixture before (red) and after (black) polymerization. The two vinyl protons of the methacrylate end group (peak **a**, δ ≈ 5.6 and 6.2 ppm, multiplet) are present only in the unreacted macromonomer and serve as an internal reference. The four methylene protons adjacent to silicon at both ends of the PDMS chain (peak **b** and **b'**, δ ≈ 0.66 ppm, multiplet) appear in both the macromonomer and the bottlebrush polymer. The conversion is thus (5.03 – 4.04)/4.04 × 100% = 25%, giving 160 × 25% = 40 PDMS side chains per bottlebrush.

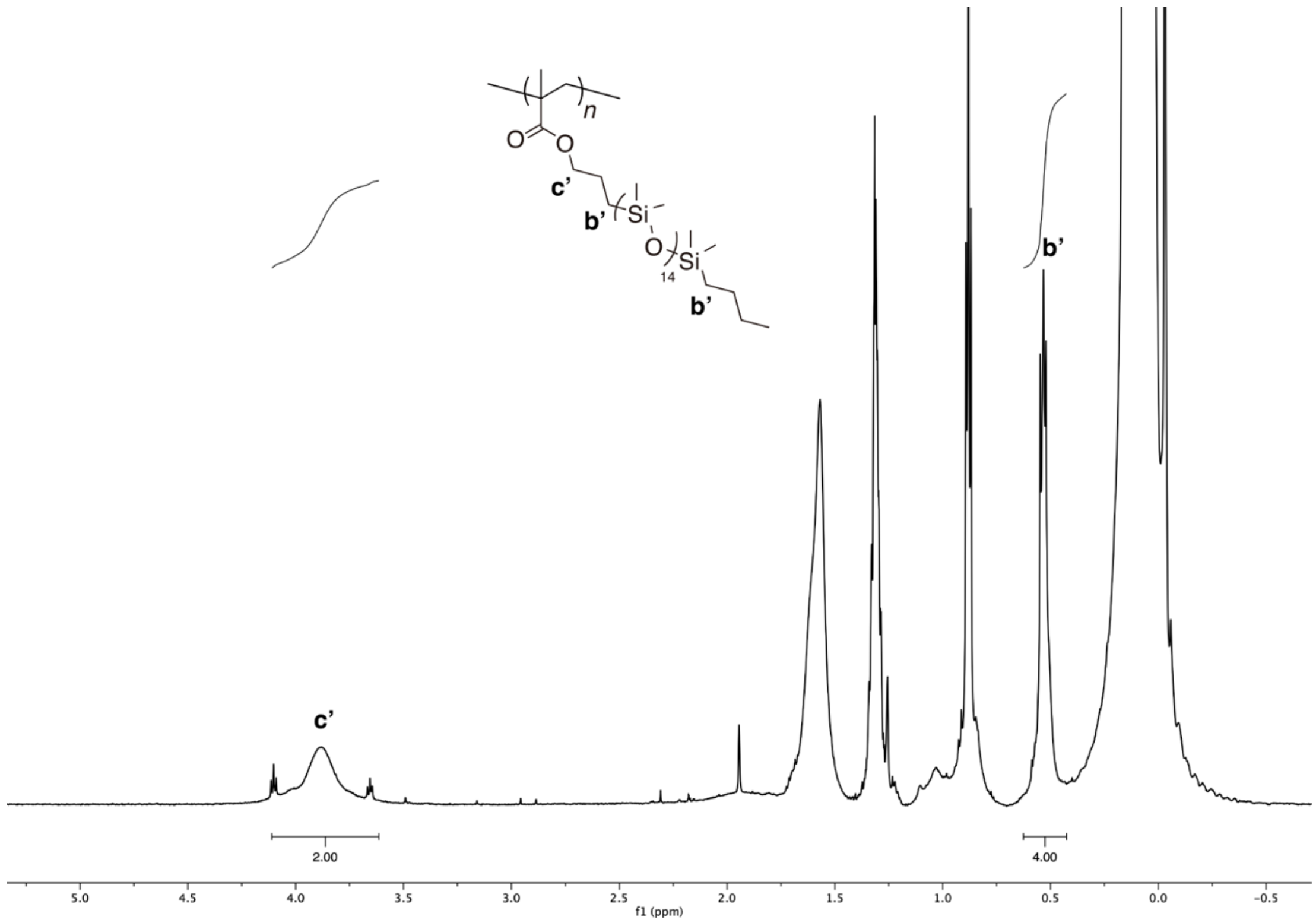

Fig. S5. ^{1}H NMR of pure $(PDMS)_{40}$ bottlebrush polymer. The two methylene protons adjacent to the methacrylate ester group (peak **c'**, multiplet, δ ≈ 3.9 ppm) and the four protons adjacent to silicon at both ends of the PDMS side chains (peak **b'**, multiplet, δ ≈ 0.5 ppm) confirm the bottlebrush structure.

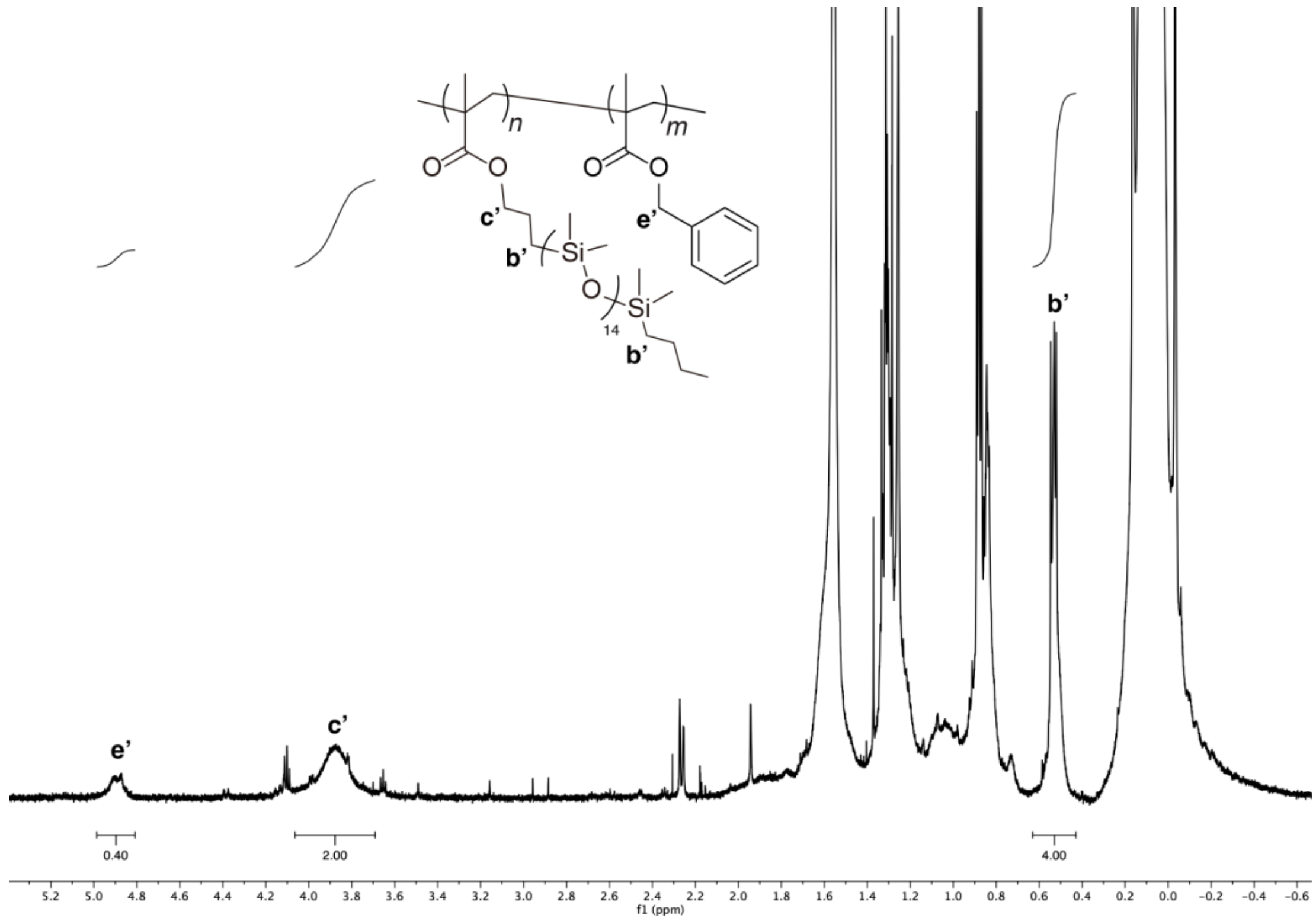

Fig. S6. ^{1}H NMR of pure $(PDMS)_{40}$-$BnMA_8$ diblock copolymer. The two methylene protons adjacent to the methacrylate ester group of the PDMS block (peak **c'**) and those adjacent to the ester group of the BnMA block (peak **e'**, multiplet, $\delta \approx 4.9$ ppm) are used to determine the degree of polymerization of the PBnMA block, DP = 40 × 0.40/2 = 8.

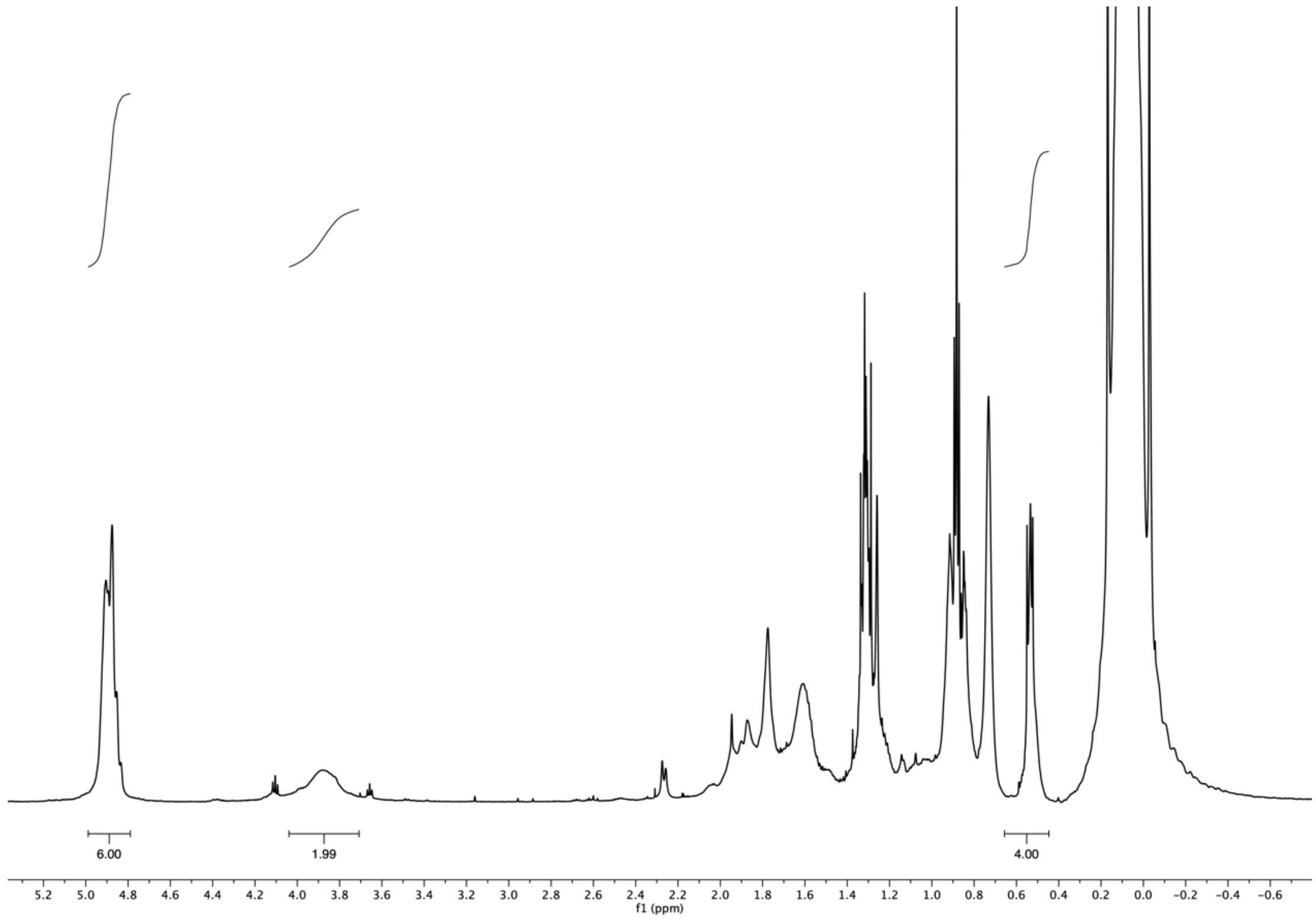

Fig. S7. ^{1}H NMR of pure $(PDMS)_{40}$-$BnMA_{120}$. DP of BnMA is 40×6.00/2=120.

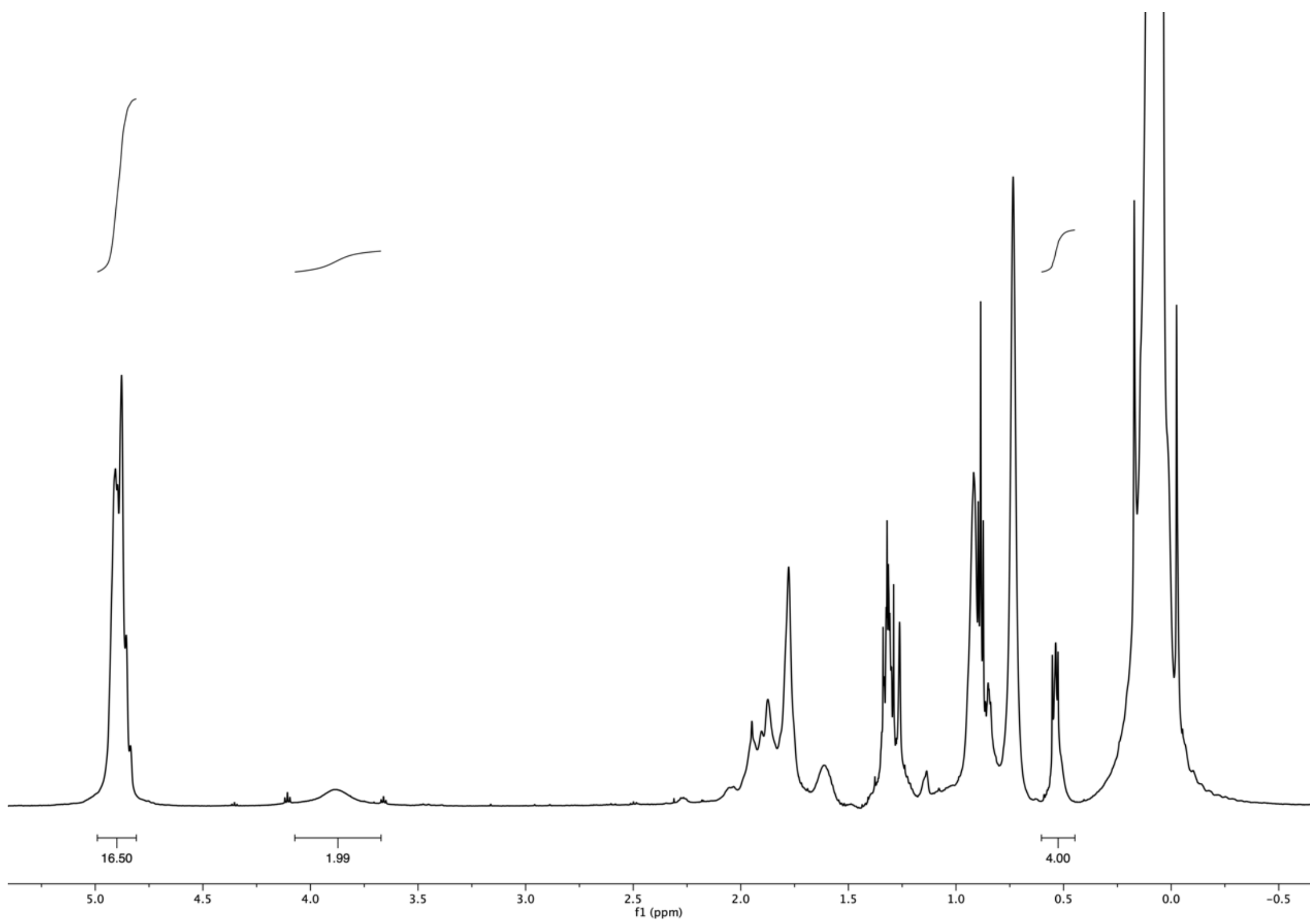

Fig. S8. ^{1}H NMR of pure $(PDMS)_{40}$-$BnMA_{330}$. DP of BnMA is 40×16.50/2=330.

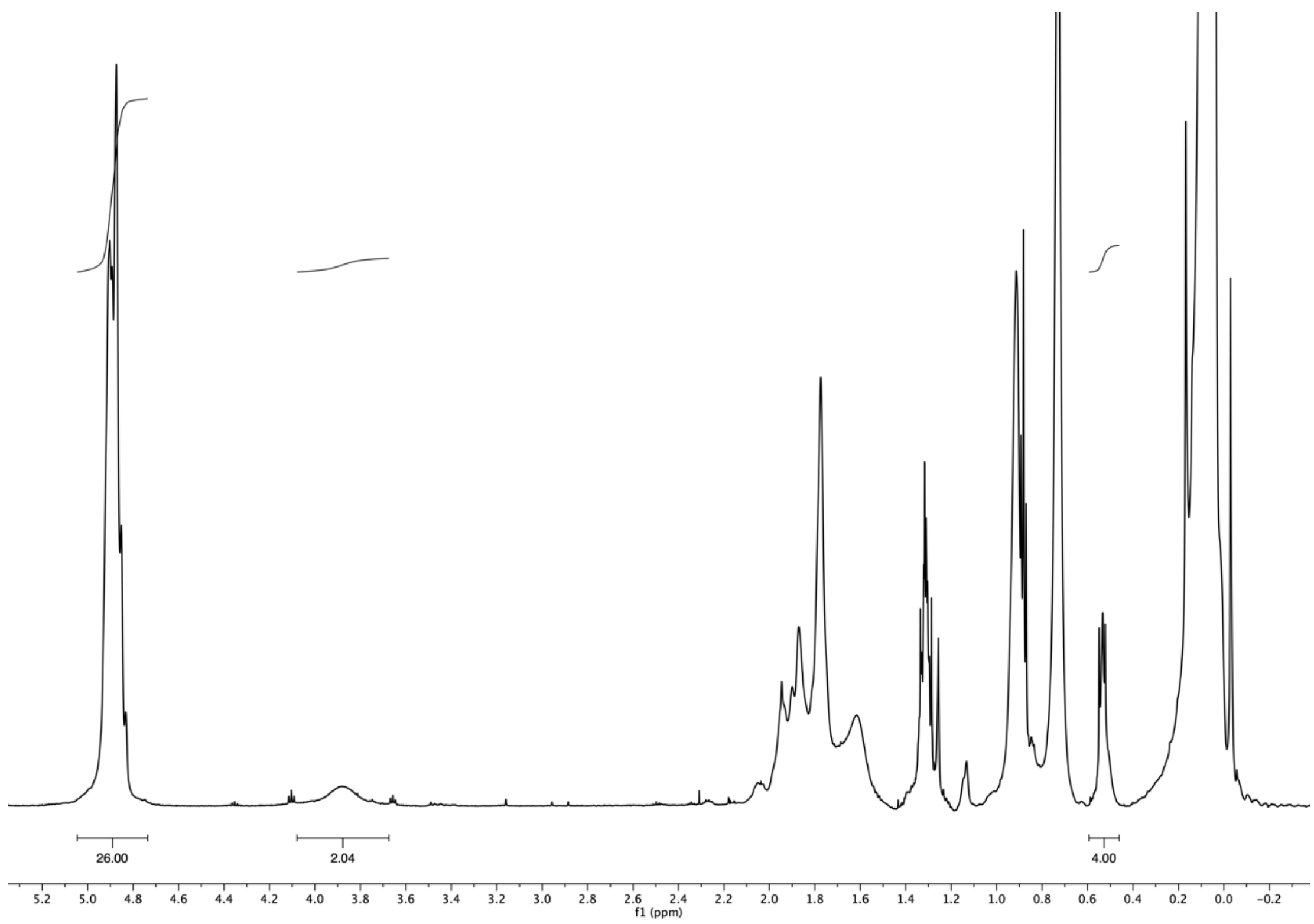

Fig. S9. ^{1}H NMR of pure $(PDMS)_{40}$-$BnMA_{520}$. DP of BnMA is 40×26/2=520.

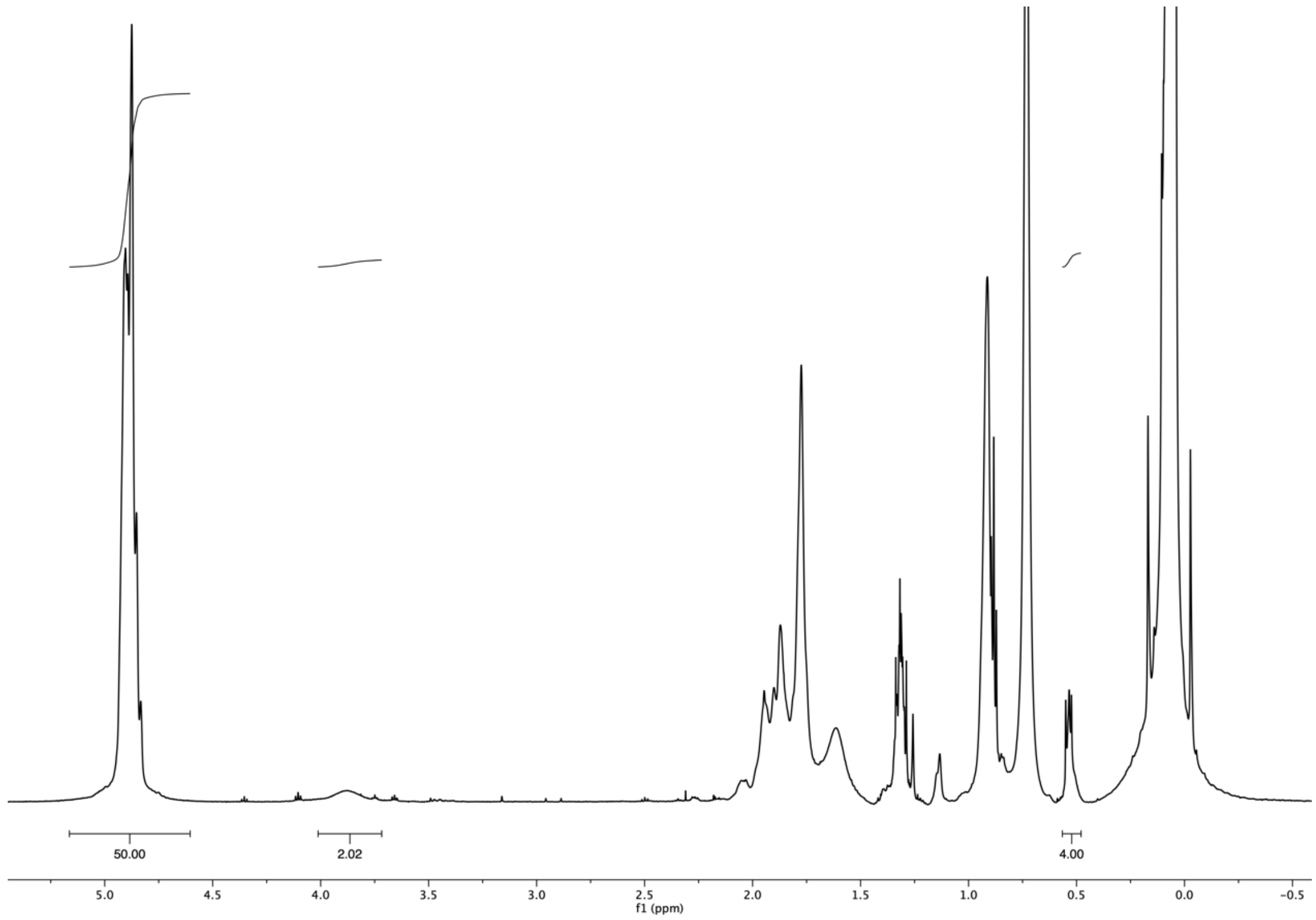

Fig. S10. ^{1}H NMR of pure $(PDMS)_{40}$-$BnMA_{1000}$. DP of BnMA is 40×50/2=1000.

References

(1) Cai, L.-H. Molecular Structure of Foldable Bottlebrush Polymers in Melts. *Macromolecules* **2025**. https://doi.org/10.1021/acs.macromol.4c02981.

(2) Paturej, J.; Sheiko, S. S.; Panyukov, S.; Rubinstein, M. Molecular Structure of Bottlebrush Polymers in Melts. *Sci. Adv.* **2016**, *2* (11). https://doi.org/10.1126/sciadv.1601478.
